# Development of Novel Eating Error Scoring Tool to Evaluate Adult Eating Behavior Anomalies Among the United Arab Emirates Population

**DOI:** 10.7759/cureus.25679

**Published:** 2022-06-05

**Authors:** Samra Abouchacra, Juma AlKaabi, Satish Chandrasekhar Nair, Abdishakur Abdulle, Mazen Taha, Mohamad Milad Ismail, Mazen Askheta, Ali El Houni, Kurady Bairy, Raghavendra Bhat, Thekra Abdul Salam Al Sayadi, Oudi Abouchacra, Durra Al Baloushi, Asma Al Nasseri, Nicole Gebran, Omar Yaman, Charu Sharma

**Affiliations:** 1 Nephrology, Al Ain Hospital, Al Ain, ARE; 2 Endocrinology, College of Medicine and Health Sciences, United Arab Emirates University, Al Ain, ARE; 3 Medical Research, Tawam Hospital, Al Ain, ARE; 4 Public Health Research Center, New York University Abu Dhabi, Abu Dhabi, ARE; 5 Gastroenterology, Tawam Hospital, Al Ain, ARE; 6 Endocrinology and Diabetes, Al Ain Hospital, Al Ain, ARE; 7 Endocrinology and Diabetes, Tawam Hospital, Al Ain, ARE; 8 Pharmacology, Ras Al Khaima Medicine and Health Sciences University, Ras Al Khaima, ARE; 9 Internal Medicine, Ras Al Khaima Medicine and Health Sciences University, Ras Al Khaima, ARE; 10 Family Medicine, Al Ain Hospital, Al Ain, ARE; 11 Chiropractic, Inspired Results, Abu Dhabi, ARE; 12 Family Medicine, Ambulatory Health Services, Al Ain, ARE; 13 Nutrition, Al Ain Hospital, Al Ain, ARE; 14 Clinical Pharmacy, SEHA Abu Dhabi Health Services, Abu Dhabi, ARE; 15 Medical Affairs, Khalifa University for Science, Technology and Research, Abu Dhabi, ARE; 16 Internal Medicine, College of Medicine and Health Sciences, United Arab Emirates University, Al Ain, ARE

**Keywords:** eating habits, behavior modification, overweight, obesity, eating behavior

## Abstract

Introduction

Maladaptive eating behaviors are emerging as the most significant determinants of obesity with a promising role in intervention. In the absence of a standardized tool to assess eating variations, an Eating Error Score (EES) tool was devised which comprised five zones for evaluating the severity of obesogenic behaviors as well as the specific area(s) with the highest susceptibility. This pilot study was aimed at evaluating the effectiveness of the EES in quantitating the eating behavior errors associated with excess weight and identifying the most affected zones.

Methods

The EES questionnaire was designed to explore potential disturbances in five zones of eating behavior related to the impetus to eat (Munger), meal choices and attentiveness to cravings (Impulsive), consumption speed (Speed feeding), cues to stop ingestion (Indulgent) and the social aspect of eating (Relationship). The questionnaire was conducted on adults with varying body mass index (BMI) attending governmental outpatient clinics. The correlation between EES and BMI was determined through Pearson Coefficient.

Results

A total of 204 participants completed the EES questionnaire. There were 72 males and 132 females with a mean BMI of 27.63 ± 6.16 kg/m^2^ and with nearly equal distribution between normal weight (37.2%), overweight (32.4%), and obese (29.4%) individuals. Nearly 75% of our cohort had a moderate total EES, and the remainder was equally distributed between the mild and severe ranges.

A weak but significant correlation was observed between total EES and BMI (r=0.275, p<0.001) suggesting increasing obesogenic styles in participants with excess weight. In addition, a similar weak but significant correlation was noted between Body Mass Index and the Munger and Impulsive zones (r=0.266 and 0.258 and p<0.001, respectively) suggesting more severe maladaptive eating behaviors in these areas. No correlation was found with the Speed feeding, Indulgent, and Relationship zones.

Conclusion

The EES may be a useful tool for assessing the extent of maladaptive eating behaviors, which predispose individuals to weight gain and sabotage their weight loss efforts. Undoubtedly, the utility of the tool needs to be corroborated in large population studies. Further, identifying the specific operant zones may show promise as many of these habits are potentially modifiable and can be targeted for weight control, most notably those associated with the Munger and Impulsive zones.

## Introduction

Obesity has become the leading lifestyle disease of the century and is responsible for a myriad of health-related ailments [1,2]. It appears that pervasive maladaptive eating behaviors are underappreciated as important factors predisposing and promoting weight gain [3,4]. Furthermore, given the non-sustainable, transient results of restrictive diets, modifications of these eating behaviors are proposed as effective interventions for weight management [3,5]. There are multiple eating behavior assessment tools that are widely utilized to uncover detrimental consumption patterns. The Eating Attitudes Test (EAT -26), for example, aims to reveal potential serious eating disorders which require professional evaluation [6]. Whereas the Three-Factor Eating Questionnaire [7] and its revised versions [8-10] assess three cognitive-behavioral dimensions namely cognitive restraint, emotional and uncontrolled eating. The latter has been extensively validated in different populations including adolescents and adults of varying body weights [9-13]. Moreover, the Adult Eating Behavior Questionnaire seeks to evaluate an imbalanced approach to food ingestion in the realms of enjoyment, responsiveness, hunger, and emotional overeating, as well as avoidance practices such as fussiness, slowness, satiety responsiveness, and emotional undereating [14]. This has also been validated in adolescent and adult populations [15,16].

Our Gulf Eatology Research Group (GERG) previously uncovered various “obesogenic” eating styles in a group of adolescents [17] as well as in overweight and obese adults from UAE; latter from unpublished data. The most significant findings were related to the following five categories namely: the impetus to eat, meal choices and attentiveness to cravings, consumption speed, cues to stop ingestion, and the social aspect of eating. An Eating Error Score (EES) was devised based on our findings, to serve as a tool for evaluating the severity of these particular maladaptive eating behaviors as well as the specific area(s) of most susceptibility. In contrast to other eating behavior questionnaires, the EES assesses one’s abilities to identify internal cues related to food intake, satiety, and cravings as well as their skill in practicing mindfulness while eating alone and countering the influences of others. The EES comprises the aforementioned five categories, which will be referred to as zones. This unique assessment tool would then potentially help in quantifying obesogenic behavior in these important areas of nutrition and identify the target areas to be addressed for successful weight control.

The aim of this pilot study was to examine the effectiveness of our EES as a tool for measuring eating behavior errors of patients with varying Body Mass Indices (BMIs), with the anticipation of uncovering more disturbed eating patterns, reflected by a higher total score, in overweight and obese persons. The secondary aim of the study was to identify, in these latter groups, behavioral susceptibilities within any of the particular zone(s).

## Materials and methods

Questionnaire design

Modeling on existing eating behavior questionnaires and using similar scoring scales, the EES questionnaire was designed based on our five identified eating error zones. The zones, defined in Table 1, were given equal weighting and each was comprised of three questions with an allocated total possible score of 3 points for that particular zone. Each question was given an empiric grade to suggest the severity of the eating error as mild (score <1), moderate (score 1-2), or severe (score >2). Total EES, which is the composite sum of the scores in each of the zones, could reach a maximum of 15 with the corresponding severity arbitrarily defined as mild (score <5), moderate (score 5-10), or severe (score >10). The higher the score in the individual zones, as well as the total EES, is interpreted to reflect a greater disturbance in maladaptive eating styles. The specific questions sought to identify the eating pattern used in the majority of one’s meals with the allocated scoring system for each of the answer options as shown in Table 1.

**Table 1:** Eating error scoring tool

Zone (Maximum Score)	Definition	Questions Comprising Relevant Zone (Score)
Munger (3)	The mental desire to eat based on external stimuli such as seeing or smelling food, which is not based on the body’s true need for fuel.	1- Decision to eat comes from: (0) one’s self, (1) others
		2- Meal consumption driven by: (0) physical hunger, (1) mental hunger (see, smell food), stress, boredom, mealtimes
		3- Location of hunger perception: i(0) in upper abdomen, (1) elsewhere in the body or no perception
Impulsive (3)	Reflex eating with self-unawareness and meal choices being based more on outside cues than inner cravings.	4- Meal choices based on: (0) cravings food palatability, (1) availability
		5- Food cravings based on: (0) internal cues, (1) external cues such as advertisement, friends, and others
		6-Taking into consideration post meal feedback from prior similar meals: (0) always, often (0.5) sometimes, (1) never
Speed feeding (3)	Tendencies towards eating quickly because of internal or external influences.	7-Typical meal consumption duration: (0) greater or equal to 20 minutes, (1) less than 20 minutes
		8-Taking time to appreciate food presentation, smell flavor texture: (0) always, often (0.5) sometimes rarely, (1) never
		9-Triggers for eating fast (rushed, stressed eating with others, very hungry, eating favorite foods): (0) do not typically eat fast, (0.5) selects two or fewer, (1) selects more than two
Indulgent (3)	Eating more than needed (overconsumption) because the pre-satiety signal indicating having had enough is missed or not perceived.	10- Stop eating triggers: (0) satisfied that had enough to last until next meal, (1) finishing plate even if full or experiencing overeating symptoms with physical discomfort
		11- Experiencing post meal symptoms: (0) none, (1) physical symptoms or guilt
		12- Reasons for overeating: (0) knowing meal has to last longer than normal (1) eating fast, influence of others, emotional (stressed, bored, sad or lonely, favorite foods)
Relationship (3)	Social influences negatively affecting the individual’s eating behavior	13- Eating to please family or others: (0) rarely, never (0.5) sometimes (1) always, often
		14- Ability to turn down food offerings: (0) always, often (0.5) sometimes (1) rarely, never
		15-Impact of eating with others: (0) same as usual eating pattern (0.5) selects one option from below (1) selects more than one option from below: a) eating when not hungry, b) eating more or quicker than usual, c) becoming non-selective with foods consumed
Total Eating Error Score (15)	(Sum of the Eating Error scores in each of the 5 Zones)	

Inclusion and exclusion criteria

The EES questionnaire is a self-administered online questionnaire available in both English and Arabic languages. Adult participants attending outpatient public health centers in the United Arab Emirates (UAE) were recruited. Included were adult patients of varying BMIs from different ethnicities who agreed to partake in our study. Patients with cognitive disabilities interfering with their ability to complete the questionnaire were excluded. Our study was conducted between 2020 and 2021 and had been approved by Al Ain Medical District Ethics Committee with approval number THREC-703. Its implementation was in compliance with all the requirements including informed consent, anonymity of patient information and data protection.

Statistical analysis

Data were analyzed using SPSS Statistical Software Version 27 (SPSS Inc. Chicago, USA). Descriptive statistics were performed, and results were represented as frequency mean standard deviation and percentages. The correlation of the dependent variable (EES) with other independent variables (BMI) was conducted using the Pearson Coefficient, for all the variables, the level of significance was taken as P<0.05.

## Results

The EES questionnaire was completed by 204 participants (72 males:132 females) as shown in Table 2. The majority of our cohort were expatriates accounting for over 75% of the entire study population. The age of our participants ranged between 16 and 65 years with an average of 38 ± 9 years, 62% of whom were married, 33% single and 5% divorced. The majority (83%) of our participants were healthy with only a few affected by chronic diseases (diabetes, hypertension, or hypercholesterolemia). The average weight was 76.75 ± 19.68 kg and height 166.41 ± 11.06 cm yielding an average BMI of 27.63 ± 6.16 kg/m^2 ^(with a minimum of 14.52 and a maximum of 54.55 kg/m^2^).

**Table 2:** Participant demographics (patient reported data)

	Frequency	Percentage (%)
Male	72	35.3%
Female	132	64.7%
UAE Nationals	50	24.5%
Others (Expat)	154	75.5%
Age	38.04 ± 9.91 yrs	
Marital Status		
Divorced	10	4.9%
Married	126	61.8%
Single	68	33.3%
Average Weight	76.75 ± 19.68 kg	
Average Height	166.41 ± 11.06 cm	
Average BMI	27.63 ± 6.16 kg/m^2^	
Comorbidities		
Diabetes	12	5.9%
High blood pressure	11	5.4%
High cholesterol	12	5.9%
None	169	82.8%

The BMI frequency distribution as well as the EES ranges as shown in Table 3 reflect a nearly even distribution between normal weight (37.2%), overweight (32.4%), and obese (29.4%) individuals with only two underweight participants (1%). The majority (72.5%) of our cohort had a moderate total EES between 5 and 10 and almost 14% in each of the mild and severe ranges.

**Table 3:** Frequency of distribution

BMI	Frequency (percentage)
<18.5 (Underweight)	2 (1%)
18.5- 24.9 (Normal)	76 (37.2%)
25.0-29.9 (Overweight)	66 (32.4%)
> 30 (Obese)	60 (29.4%)
Eating Error Score	
<5 (Mild)	28 (13.8%)
5-10 (Moderate)	148 (72.5%)
>10 (Severe)	28 (13.7%)

As shown in Table 4, there was a weak but significant correlation between total EES and BMI with a Pearson Coefficient of 0.275 and a highly significant p-value (p<0.001). This suggests that with increasing BMI, the individuals tend to have a higher total EES signifying they possess more pronounced obesogenic behaviors. Similarly, there was also a weak but significant correlation between BMI and the Munger Zone score as well as the impulsive zone score (Pearson Coefficient 0.266 and 0.258, respectively, with a highly significant p-value (p<0.001). This again suggests that with higher BMIs, individuals have more pronounced maladaptive eating behaviors related to the Munger and impulsive areas of their nutrition. There was no significant correlation between BMI and speed feeding, indulgent and relationship zones, respectively.

**Table 4:** Correlation of total score and zones score with BMI using Pearson coefficient * P<0.05; ** P<0.01

		Pearson correlation	95% CI	P-value
BMI	Munger Zone	0.266**	0.133-0.389	< 0.01
	Impulsive Zone	0.258**	0.125-0.382	< 0.01
	Speed Feeding Zone	-0.004	-0.142-0.133	0.950
	Indulgent Zone	0.145*	0.007-0.277	0.039
	Relationship Zone	0.115	-0.023-0.248	0.103
	Total EE Score	0.275**	0.143-0.397	<0.01

## Discussion

Our earlier studies indicated that with respect to the impetus to eat, only one-third of our cohorts perceived the appropriate hunger sensation with the decision to eat in a significant proportion not being based on the actual need for food [17]. Rather it originated from outside influences, boredom, stress, or according to pre-set mealtimes. When it came to meal choices and attentiveness to cravings, the majority of our participants mostly consumed based on food palatability and availability with less than one-fourth basing the decisions on their own cravings. In addition, cravings in our cohort were attributed to inner physical or psychological desires in 64% of adolescents but only 47% of adults. The latter is mainly influenced by external stimuli based on advertising or peer effects.

Importantly, in addition to excessive food quantities, high speed of food intake was observed with meal consumption in under 20 minutes in 75% of adolescents and 85% of adults. Moreover, 46% of adults reported even more accelerated eating speeds in under 10 minutes, which was also seen in almost one-fifth of adolescents. On the other hand, speed feeding triggers in our cohorts were excessive hunger and being rushed reported in nearly two-third of both groups. Furthermore, our results uncovered that the foods that are consumed quickly in over 80% of adolescents and adults, were favorite foods, raising the possibility that excitement, may be an important speed feeding trigger. With regards to meal conclusion, 20% of adolescents described that they would typically stop eating when they finished their plate, or experienced symptoms of overfullness namely could not breathe, get off the chair, or had to unbuckle their belt; in line with the frequent subclinical binging reported in adolescents. These observations were more pronounced in adults reaching 36% and may perhaps suggest inattention or failure recognize the innate cues signaling satiety, hence leading to overeating. Post-meal symptoms were reported in a similar and significant proportion of both groups, with 81% of adolescents and 75% of adults experiencing physical as well as 22% of adolescents and 52% of adults struggling with post-meal guilt on regular basis. Interestingly, 36% of adolescents and 52% of adults practically disregarded altogether symptomatic feedback for future eating decisions. This is surprising since meal dissatisfaction was conveyed in nearly half of our adult cohort, which would have been expected to instigate a behavioral change.

The social aspect of eating also revealed interesting findings whereby eating to please their family was prevalent in 53% of adults and was even more pronounced in adolescents reported in 78%. Likewise, eating to please others was significant but similar in both groups (48% adolescents vs 44% adults). More importantly, 44% of adolescents described an inability to refuse food offered by their family with an even higher percentage in adults reaching 61%. Adolescents, however, were more adept at overcoming social pressures to eat from other persons, with only 28% succumbing to their food offerings versus 59% of adults.

Our data suggest that the Eating Error scoring methodology might be helpful in assessing maladaptive eating behaviors since the total score appears to be positively correlated with BMI. This would imply a tendency for overweight and obese individuals to possess more obesogenic eating behaviors which would then be quantifiable using this tool. Although the correlation was weak, it was still highly statistically significant perhaps suggesting that it might be due to the small sample size. Consequently, the component zones comprising the total EES would be expected to have an instrumental role in promoting weight accumulation. However, only the Munger and Impulsive zones showed a positive correlation with increasing BMI which could similarly allow quantitation of the severity of maladaptive eating styles specifically related to these zones.

The questions in the Munger zone aimed to assess the individual’s ability to recognize and act upon proper hunger cues. An underdeveloped awareness or attention to hunger would tend to suggest that food consumption is not based on the physiological need for fuel but rather according to “mental” hunger or external cues outside oneself. The latter would include ingestion decisions based on influences of others, set mealtimes, stress, or boredom. Mental hunger implies the desire to eat based on seeing or smelling food, which is not based on a need for refueling and hence promoting weight gain. Interestingly, the heightened awareness of this primitive yet powerful hunger signal can be traced to the newborn stage where feeding timings are solely dependent on such physiological input. It is possible that over time, this awareness might become attenuated or disregarded. Although this correlation was found to be weak, its high statistical significance likely indicates the need for a larger sample size to definitively show such a relation.

The inquiries in the Impulsive zone were meant to demonstrate the ability to identify and act upon true internal cravings, as opposed to susceptibly to external cues. In addition, it was also intended to reveal whether sufficient attention is paid to post-meal feedback for future meal decisions. This is because lack of attention to internal cravings could play an integral role in overeating since cravings are very common. Catering to emotional needs must be specifically matched to be satisfied [18,19], and if suppressed may lead to overindulgence [20]. Our observed correlation of the Impulsive zone with BMI would suggest an important obesogenic behavior where food choices are based on outside cues such as food availability and palatability rather than internal cravings. These external factors particularly food taste have been reported as the most significant determinants of item selection [21,22]; a principle exploited by the food industry through additives and flavor enhancers. In addition, it would also signify that cravings themselves are influenced by external factors namely advertisement or the effect of others. Furthermore, it would indicate that insufficient attention is given to post-meal feedback from prior similar meals which could lead to post-meal symptoms and meal dissatisfaction as observed in our adult and adolescent studies. This was also reported by Monrroy where strong and persistent unpleasant digestive sensations occurred after a probe meal in adults in addition to emotional symptoms which were more pronounced in women [23]. Similar findings have been described in adolescents with manifestations of overeating, subclinical binging, and associated psychological symptoms being experienced after consumption [24,25].

With respect to the remaining three zones, the lack of observed correlation does not necessarily diminish their importance as obesogenic behaviors, and may in fact be secondary to our study being underpowered because of the small sample size. This is especially true given their observed importance in the literature as will be discussed. In terms of speed feeding, an association has been reported between the rapid rate of eating, overconsumption, and overweight [26,27]; perhaps because of missing the satiety awareness. This complex process involves neuro-humoral signaling from the stomach to the central nervous system indicating the cue for intake cessation [28,29], which typically takes 20 minutes from the start of a meal. The significance of speed feeding becomes even more important in the context of social mirroring whereby modeling has also been observed on the consumption speed of others [30,31]. In fact, decelerating food intake speed through mindfulness has been advocated as a means for effective weight management [32]. Erlanson-Albertsson also observed that when hunger signals are too strong this leads to overconsumption [33] perhaps again through the speed feeding pathway.

Additionally, for the Indulgent zone, it has been observed that when satiety signs to stop eating are too weak this leads to overeating likely because the individual is then completely dependent on external cues for ending their consumption. These factors could include ingestion cessation when everyone has stopped eating or the classic “finishing the plate” phenomenon; the latter was reported by 14% of adolescents and 26% of adults [34,35].

With respect to the Relationship zone, the importance of external influences on eating behavior has been well described in adolescents [22] underlining a key role for modeling on their family and peers [35]. Importantly, the parental impact appears to be much stronger [22] and is a better predictor of eating behaviors in adolescents [36]; presenting a promising opportunity for influencing healthy consumption in children. In adults also, there is strong evidence for modeling eating styles especially when norms are observed as relevant [37] irrespective of the person’s hunger or dieting goals [38]. Some individuals known as “people pleasers” tend to consume and even overeat only to please others [39]. Facilitation is another mechanism whereby social stimuli can influence food intake by encouraging more consumption when eating with others [40]. All of this highlight the importance of focusing on internal cues in order for the individual not to succumb to social pressures to consume. Clearly, these other zones may be equally important obesogenic behaviors and serious contributors to weight gain.

Since all the EES maladaptive behaviors lend themselves well to behavior modification, focused attention on the zones more severely affected, is anticipated to positively impact intake moderation and weight control efforts [17]. For instance, awareness training would be beneficial, for the recognition and acting upon the satiety cues as well as cravings. The latter enables individuals to combat external influences without falling victim to marketing ploys. Additionally, addressing personal behavior changes such as attentiveness to internal cues for consumption based on the need for fuel as well as decelerating the eating speed [26], reduction of the quantity of food consumed, and acquiring an appreciation for post-meal feedback could be equally effective.

## Conclusions

This study demonstrated the ability of the EES to uncover more disturbed eating patterns in overweight and obese individuals, suggesting it could be an effective tool for quantifying maladaptive behaviors as well as the specific zones with more obesogenic trends. Their identification would help individuals navigate the complex journey of weight loss where focus can be given to the areas of most serious susceptibility. The Munger and Impulsive zones appear to be areas of particular importance and likely high yield in focused weight loss efforts.

It is anticipated that larger studies are required to validate and strengthen our preliminary data. The utility of quantitating disturbances in individual eating styles through the EES, may not be limited to our local population. In fact, it might be generalizable for assessment of maladaptive eating patterns and unveiling the areas of most concern in other cultures, given the high proportion of expatriates in our cohort. Recognition and addressing of detrimental eating behaviors presents the best opportunity to combat the obesity pandemic and its usefulness applies to the overweight and obese in addition to normal weight individuals and children as a preventative measure. Our study attempts to unearth anomalies in eating behavior using the EES tool, despite the limitations contributed by the low sample size, the strength of the study is that the EES tool enables targeting of interventions at the critical stages of eating behavior.
